# Anti-*Klebsiella pneumoniae* activity of secondary metabolism of *Achromobacter* from the intestine of *Periplaneta americana*

**DOI:** 10.1186/s12866-023-02909-7

**Published:** 2023-06-05

**Authors:** Yan Ma, Ping Guo, Xueqin Chen, Minhua Xu, Wenbin Liu, Xiaobao Jin

**Affiliations:** 1 School of Life Sciences and Biopharmaceutics, Guangdong Pharmaceutical University, Guangzhou, 510006 China; 2 Guangdong Provincial Key Laboratory of Pharmaceutical Bioactive Substances, Guangdong Pharmaceutical University, Guangzhou, 510006 China; 3 Clinical Laboratory, Shenzhen Bao’An District Central Hospital, Shenzhen, 518103 China; 4 Clinical laboratory, Foshan Fosun Chancheng Hospital, Foshan, 528000 China

**Keywords:** *Klebsiella pneumonia*, *Periplaneta americana* intestine, *Achromobacter*, Secondary metabolites, Antibacterial activity

## Abstract

**Background:**

*Klebsiella pneumoniae* is one of the main pathogens of clinical isolation and nosocomial infections, as *K. pneumoniae* show broad-spectrum resistance to β-lactam and carbapenem antibiotics. It is emerging clinical need for a safe and effective drug to anti-*K. pneumoniae*. At present, *Achromobacter* mainly focused on its degradation of petroleum hydrocarbons, polycyclic aromatic hydrocarbons, assisting insects to decompose, degrade heavy metals and utilize organic matter, but there were few reports on the antibacterial activity of the secondary metabolites of *Achromobacter*.

**Results:**

In this study, a strain WA5-4-31 from the intestinal tract of *Periplaneta americana* exhibited strong activity against *K. Pneumoniae* through preliminary screening. The strain was determined to be *Achromobacter sp.* through the morphological characteristics, genotyping and phylogenetic tree analysis, which is homologous to *Achromobacter ruhlandii* by 99%, its accession numbe in GenBank at National Center for Biotechnology Information (NCBI) is MN007235, and its deposit number was GDMCC NO.1.2520. Six compounds (Actinomycin D, Actinomycin X2, Collismycin A, Citrinin, Neoechinulin A and Cytochalasin E) were isolated and determined by activity tracking, chemical separation, nuclear magnetic resonance (NMR) and mass spectrometry (MS) analysis. Among them, Actinomycin D, Actinomycin X2, Collismycin A, Citrinin and Cytochalasin E showed a good effect on anti-*K. pneumoniae*, with MIC values of 16–64 µg/mL.

**Conclusions:**

The study reported *Achromobacter*, which was from the intestinal tract of *Periplaneta americana* with the activity against *K. Pneumoniae*, can produce antibacterial compounds for the first time. It lays the foundation for development of secondary metabolites of insect intestinal microorganisms.

**Supplementary Information:**

The online version contains supplementary material available at 10.1186/s12866-023-02909-7.

## Introduction

*Klebsiella pneumoniae* is recognized as a conditioned pathogen that colonizes the mucosal surface of patients and do not cause illness, but *K. pneumoniae* from the mucosa may spread to other tissues, leading to life-threatening infections and a variety of diseases, including pneumonia, urinary tract infections, blood infections, and sepsis [1, 2]. These infections are particularly prominent in newborns, the elderly, and individuals with weakened immune functions [3]. *K. pneumoniae* also causes a large number of community-acquired infections. The main feature of these infections is that their morbidity and mortality are related to carbapenem-resistant resistance of *K. pneumoniae* [4, 5]. Therefore, its emerging clinical need for a safe and effective drug anti-*K. pneumoniae.*

At present, *Achromobacter* is mainly isolated from the soil, plants and the digestive system of insects [6–9]. The research on *Achromobacter* at home and abroad mainly focused on its degradation of petroleum hydrocarbons, polycyclic aromatic hydrocarbons and synthetic new lipopeptide surfactants in petroleum-contaminated soil, assisting insects to decompose, degrade heavy metals and utilize organic matter [10–14], but there were few reports on the antibacterial activity of the secondary metabolites of *Achromobacter.* CHEN Zhiyu [15] isolated and identified four *Achromobacter* from the *Periplaneta american* intestinal. The four strains had inhibition ability to *B. subtilis, S. aureus, A. niger* and other pathogenic bacteria, and were both been detected the key secondary metabolite biosynthesis gene PKS I and NRPS. This revealed that the secondary metabolites of *Achromobacter* might have the ability to produce the anti-bacteria and anti fungi substances.

*Periplaneta americana* is a traditional Chinese medicinal material [16]. Studies had showed that the dried worms of *Periplaneta americana* had anti-inflammatory and anti-infective effects [17]. It can also promote angiogenesis, accelerate the shedding of necrotic tissues, and create conditions for tissue repair [18, 19]. In our previous studies, 159 strains of intestinal bacteria were isolated from the *Periplaneta americana* and produced a variety of antibacterial, antifungal, and anti-tumor metabolites [20–23]. In this study, a strain WA5-4-31 from the *Periplaneta americana* intestine, which exhibited strong activity against *K. Pneumoniae* through preliminary screening, was determined to be *Achromobacter* through the morphological characteristics, genotyping and phylogenetic tree analysis. The secondary metabolites of the strain WA5-4-31 were isolated and determined by activity tracking, chemical separation, nuclear magnetic resonance (NMR) and mass spectrometry (MS) analysis. This study aimed to research the material basis of the secondary metabolites of the *Achromobacter* against *Klebsiella pneumonia*. It lays the foundation for development of secondary metabolites of insect intestinal microorganisms.

## Results

### Screening and identification of strain WA5-4-31

**Fig. 1:**
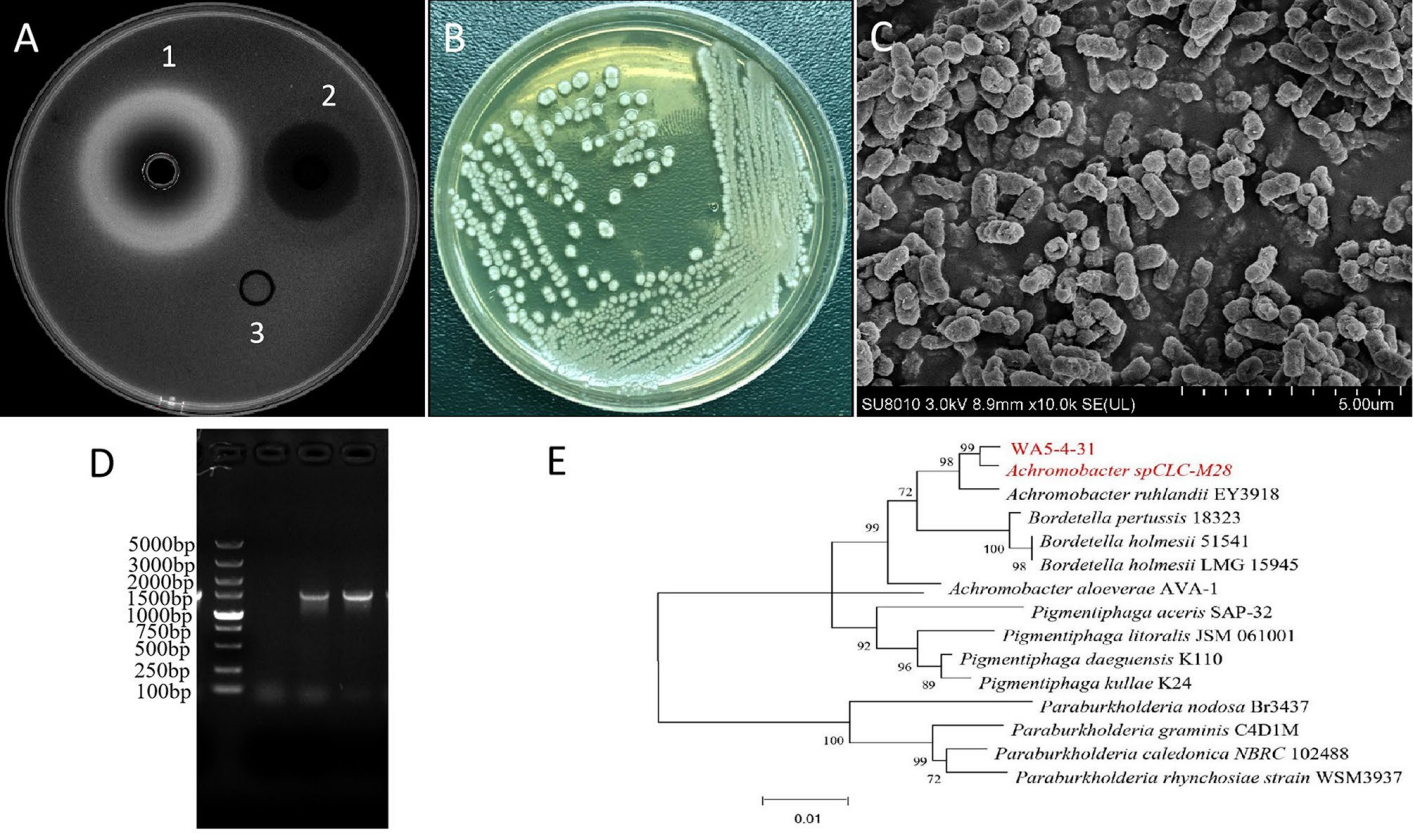
**Anti-*K. pneumonia*Activity and Identification of Strain WA5-4-31**. (**A**) The Anti-*K. pneumonia* activity of WA5-4-31, 1–3 refers to sample (EtoAc 1.024 mg/mL, 150µL), positive control (Ciprofloxacin 8 µg/mL, 150 µl ) and blank control (methanol solvent, 50 µl). (**B**) Culture on Gauze’s medium No.1. (**C**) Observation of the culture by scanning electron microscopy (10,000×). (**D**) PCR products of strain WA5-4-31 16 S rDNA by electrophoresis. (**E**) The phylogenetic tree of strain WA5-4-31. Maximum-likelihood phylogenetic tree based on 16 S rDNA gene sequences

According to the preliminary activity screening of different intestinal bacteria from *Periplaneta americana*, the secondary metabolite of strain WA5-4-31 had best anti-*K. pneumonia* activity (Fig. 1A). The colony of the strain was rounded and the surface was dry and opaque on the Gauze’s medium No.1 (Fig. 1B). Scanning electron microscopy shows that the strain was coryneform bacterium (Fig. 1C). Electrophoresis showed that the length of the target band was about 1500 bp, which was identical to the expected length (Fig. 1D).The strain WA5-4-31 is homologous to *Achromobacter ruhlandii* by 99% (Fig. 1E). The accession number of WA5-4-31 16 S rRNA was MN007235. The strain WA5-4-31 was finally determined to be of the genus *Achromobacter*, and its deposit number was GDMCC NO.1.2520.

### Isolation and Purification of Anti-***K. Pneumoniae*** Compounds

**Fig. 2:**
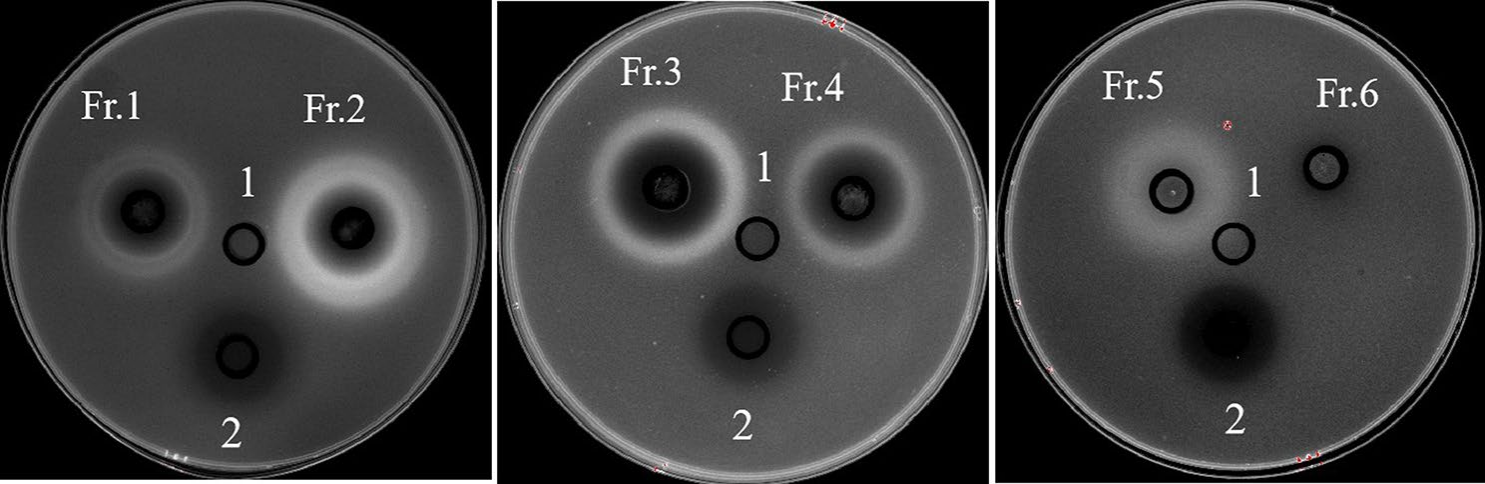
**The antibacterial activity of different fractions against*K. pneumoniae*by Oxford Cup method**. 1: Blank control: methanol solution; 2: Positive drug: Ciprofloxacin, 4 µg/mL; Fr.1- Fr.6: Methanol solution with diferent fractions, 5 mg/mL

The crude extract (18.0 g) of strain WA5-4-31was loaded onto a silica gel column and eluted with an increasing polarity of ethyl acetate (dichloromethane: ethyl acetate (1-100%)), finally nine different fractions were obtained. Eight fractions showed activity against *K. pneumonia* ATCC 13,883 by Oxford Cup method, especially the fraction 2 and 3 were better, the diameters of inhibition zones of which were 25.43 ± 0.22 and 23.61 ± 0.34. There six pure compounds were purified from the active fractions (Table 1; Fig. 2).

**Table 1:** Anti-*K. pneumoniae* activity and purified compounds of different fractions

No.	Diameter of the inhibition zone (mm) (n = 3, means ± SD)	purified compound	No.	Diameter of the inhibition zone (mm) (n = 3, means ± SD)	purified compound
Fr.1	18.52 ± 0.20	compound 1 (37.52 mg)	Fr.6	8.31 ± 0.50	
Fr.2	25.43 ± 0.22	compound 2 (36.87 mg)	Fr.7	9.15 ± 0.18	
Fr.3	23.61 ± 0.34	compound 3 (71.87 mg) compound 6 (54.23 mg)	Fr.8	8.75 ± 0.23	
Fr.4	18.50 ± 0.51	compound 5 (58.72 mg)	Fr.9	Null	
Fr.5	17.12 ± 0.54	compound 4 (28.41 mg)	Positive drug	14.68 ± 0.47	

### Spectral analysis

**Fig. 3:**
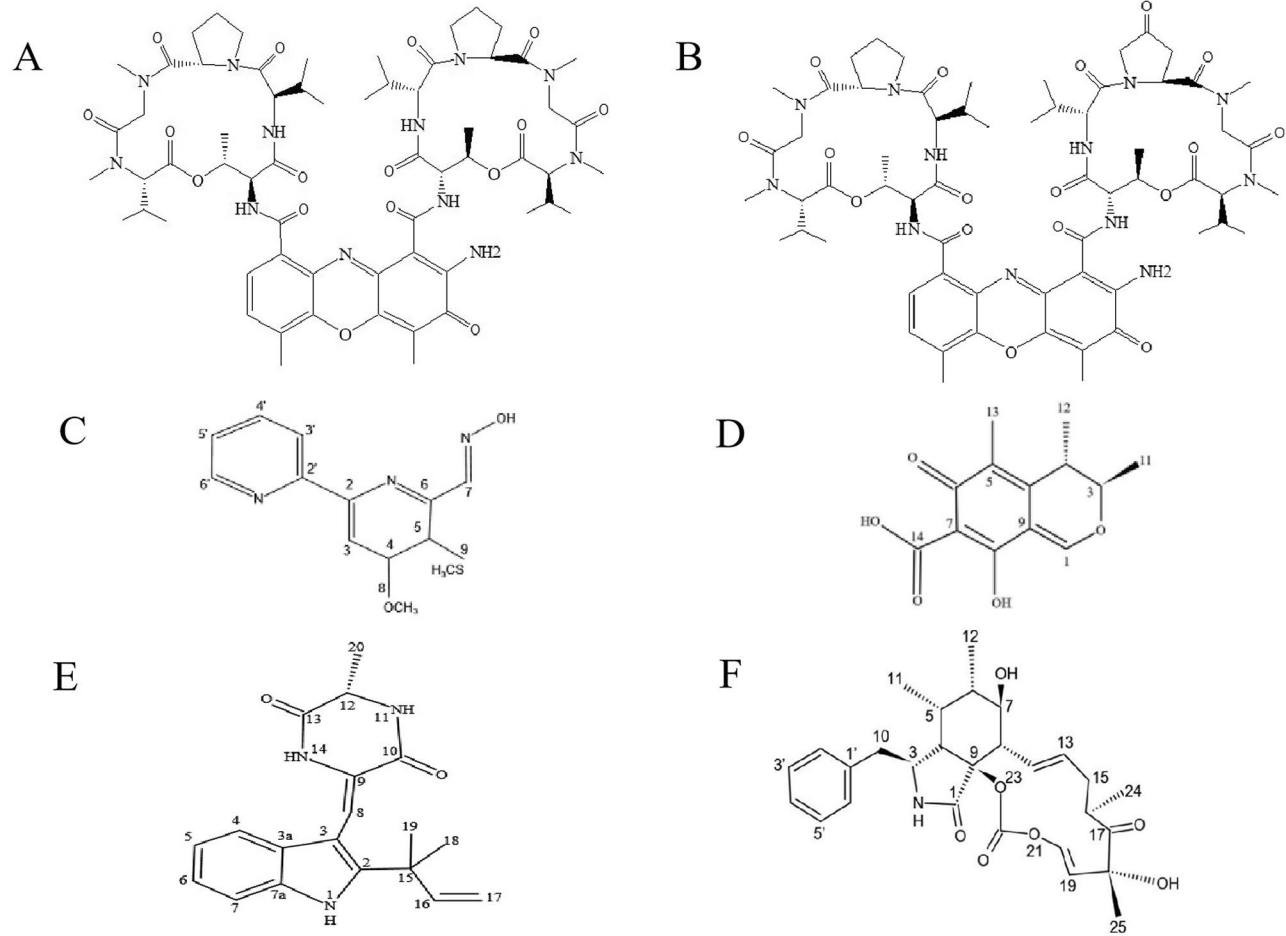
**Molecular structure of the purified compound 1–6**. (**A**) compound **1**: Actinomycin D. (**B**) compound **2**: Actinomycin X_2_. (**C**) compound **3**: Collismycin A. (**D**) compound **4**: Citrinin. (**E**) compound **5**: Neoechinulin A. (**F**) compound **6**: Cytochalasin E

Through high-resolution electrospray ionization mass spectrometry (HRESIMS) and NMR analysis and by comparison with previously reported MS and NMR data, the structures of the six pure compounds were clarified. The molecular ion peak of the mass spectrum detected by compound 1 is m/z1255.64[M + H]+, the compound 2 is m/z 1269.62[M + H]+, the compound 3 is m/z276.08[M + H]+, the compound 4 is m/z251.9[M + H]+, the compound 5 is m/z324.17[M + H]+, the compound 6 is m/z494.22[M + H]+, combined with H1 and C13 NMR spectra showed very similar to the previously reported data, so it could be determined that compounds **1**–**6** were Actinomycin D (**1**), Actinomycin X2 (**2**), Collismycin A(**3**), Citrinin (**4**), Neoechinulin A (**5**) and cytochalasin E (**6**) (Fig. 3).

### Anti-***K. pneumonia*** activity of the purified compounds

**Fig. 4:**
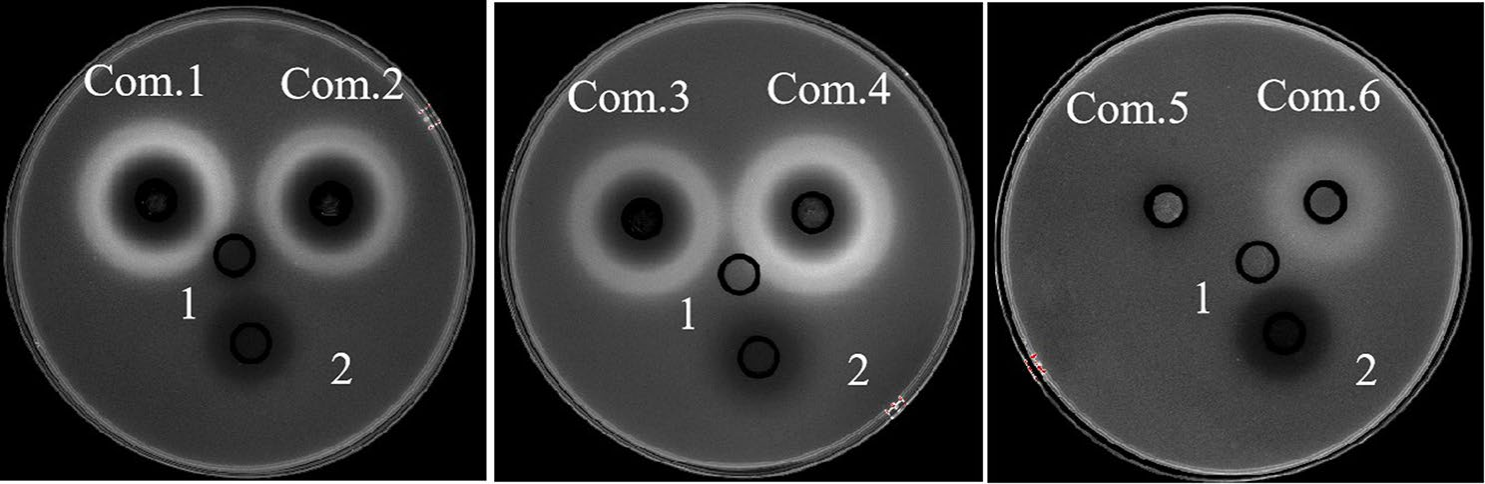
**The antibacterial activity of the purified compounds against*K. pneumoniae*by the Oxford Cup method**. 1: Blank control: methanol solution; 2: Positive drug: Ciprofloxacin, 4 µg/mL; Com.1- Com.6: the purified compounds 1–6, 1 mg/mL

**Fig. 5:**
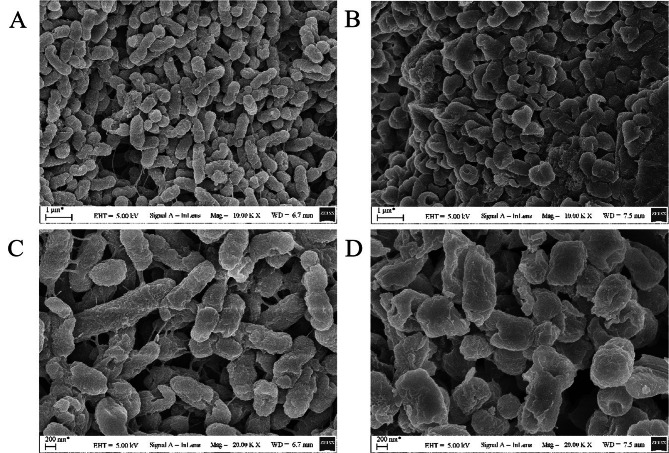
**Scanning electron microscopy (SEM) images of*K. pneumoniae*with CollismycinA treated or untreated**. (**A**) Untreated *K. pneumoniae* (10 K×). (**B**) treated *K. pneumoniae* (10 K×). (**C**) Untreated *K. pneumoniae* (20 K×). (**D**) treated *K. pneumoniae* (20 K×)

As the MIC results showed in Table 2, compound 1(Actinomycin D), compound 3 (Collismycin A) and compound 6 (Cytochalasin E) showed strong activity against *K. pneumoniae* ATCC 13,883, with MIC values of 64 µg/mL, 16 µg/mL, and 32 µg/mL. The results of the Oxford cup method showed that compound 2(Actinomycin X2) and compound 4 (Citrinin) also had a strong anti-*Klebsiella pneumonia* activity with an average inhibition zone diameter of 26.83 ± 0.52 mm, 31.00 ± 1.12 mm (Fig. 4; Table 3). SEM results showed that the cell membrane of *K. pneumoniae* was destroyed after 24 h of treatment with compound 3 (Collismycin A) (Fig. 5) .

**Table 2:** The Minimal inhibitory concentration (MIC) of Compounds against *K. pneumoniae* (MIC, µg/mL, n = 3)

MIC (mL)	Compound 1 Actinomycin D	Compound 2 Actinomycin X2	Compound 3 CollismycinA	Compound 4 Citrinin	Compound 5 Neoechinulin A	Compound 6 Cytochalasin E	positive control
K. *Pneumoniae* (ATCC13883)	64	128	16	128	-	32	0.5
S. *aureus* (ATCC25923)	128	64	2	64	-	-	32

**Table 3:** Anti-*K. pneumoniae* activity of purified compounds

No.	Diameter of the inhibition zone (mm) (n = 3, means ± SD)	No.	Diameter of the inhibition zone (mm) (n = 3, means ± SD)
Compound 1 Actinomycin D	27.87 ± 0.90	Compound 5 Neoechinulin A	
Compound 2 Actinomycin X2	26.83 ± 0.52	Compound 6 Cytochalasin E	16.57.61 ± 0.46
Compound 3 CollismycinA	27.13 ± 1.69	positive control	12.67 ± 0.68
Compound 4 Citrinin	31.00 ± 1.12		

## Discussion

With the overmining of actinomycetes for compounds acting, recent efforts to discover novel antibiotics have been focused on other groups of bacteria [24]. *Achromobacter sp.* is a short, Gram-negative rod, which occur in water, soils and the intestinal tracks of animals, can produce a variety of biologically active substances [25–27]. The insect-associated *Achromobacter sp.* is another promising system for the discovery of antibacterial and antifungal substances. Deepa I [28] found that An *Achromobacter sp.* associated with a Rhabditis entomopathogenic nematode (EPN), displayed promising antibacterial property. Three different cyclic dipeptides (CDPs), cyclo(D-Leu-D-Arg) (1), cyclo(L-Trp-L-Arg) (2), and cyclo(D-Trp-D-Arg) (3) were purified from its ethyl acetate extract by silica gel column chromatography and had active against wound associated bacteria. In a recent literature, *Photorhabdus*, gut symbionts of enthomopathogenic nematodes that carry up to 40 biosynthetic gene clusters coding for secondary metabolites, is attractive producers of secondary metabolites. This resulted in the isolation of a novel antimicrobial, 3’-amino 3’-deoxyguanosine (ADG), active against *E. coli* [29].

In the present study, we found that the secondary metabolites of the strain WA5-4-31 has anti-*K. pneumonia* activity, which isolated from the intestine of *Periplaneta americana*. We identify preliminarily the strain WA5-4-31 as *Achromobacter* through morphology and molecular biology. Six compounds (Actinomycin D, Actinomycin X2, Collismycin A, Citrinin, Neoechinulin A and Cytochalasin E) were isolated and determined from the secondary metabolites of the strain WA5-4-31, by activity tracking, chemical separation, nuclear magnetic resonance (NMR) and mass spectrometry (MS) analysis. Those compounds have been previously reported as natural bioactive secondary metabolites from *Streptomyces species* or fungi species [20, 30–32]. Till dated those compounds are reported here for the first time from *Achromobacter sp.* Moreover, this is also the first report on *Achromobacter sp.*, which can produce active compounds from *Periplaneta american* intestine.

In the previous literature, Actinomycin D, Actinomycin X2, Collismycin A, Citrinin and cytochalasin E were effective biologically active substances, which can be used to treat a variety of human diseases, such as cancer, bacterial infections, et [33, 34]. Actinomycin D inhibited acute myeloid leukemia (AML) through targeting of an oncogenic mutant form of the nucleolar chaperone nucleophosmin 1 [35]. Actinomycin D, a potent anti-virulence agent, is a promising candidate to treat *Pseudomonas aeruginosa* infection by interfering with the quorum sensing (QS) systems [36]. One strain *Streptomyces globisporus* isolated from the intestinal tract of *Periplaneta americana*, produced actinomycin X2 and collismycin A which showed strong inhibition of MRSA. Citrinin is produced by fungi of the genera *Monascus* and *Penicillium*, which displayed excellent biological activities against some pathogenic bacteria with MIC of from 4 to 16 µg/ml [37]. Cytochalasin E, a common fungal metabolite, has anti-proliferative activity against human HT-29 colorectal cancer cells [38]. Our further research showed that, Actinomycin D, Collismycin A, Citrinin and Cytochalasin E, isolated from secondary metabolism of *Achromobacter* from the intestine of *Periplaneta americana*, showed strong activity against *K. pneumoniae* with MIC values of 16 ~ 64 µg/mL. *Achromobacter* has not been reported to produce any anti-*K. pneumoniae* substances previously.

## Conclusions

Six antimicrobial compounds were isolated from the secondary metabolites of the *Achromobacter*, which has anti-*Klebsiella pneumonia* activity. Especially Actinomycin D, Collismycin A and Cytochalasin E showed strong activity against *Klebsiella pneumoniae*. Collismycin A had a destructive effect on the cell membrane of *Klebsiella pneumoniae*. This is the first reported *Achromobacter sp.*, which isolated from the intestinal tract of *P.americana*, can produce active compounds. Moreover, *Achromobacter sp*. has not been reported to produce any anti-*Klebsiella pneumoniae* substances previously, such as actinomycin D, collismycin A and Cytochalasin E. However, the activity of those compounds against some clinically relevant pathogens, such as resistant *K. pneumoniae* were still unclear. Moreover, the Whole Genome Sequencing(WGS) of the strain may need further studies.

## Materials and methods

### Microorganisms, Chemicals and Media

The Strain WA5-4-31 was isolated from the intestinal tract of *Periplaneta americana* and deposited in Guangdong Microbial Culture Collection Center (GDMCC NO.1.2520). *Klebsiella pneumoniae (*ATCC13883), *Staphylococcus aureus* (ATCC25923) were obtained from Guangdong Institute of Microbiology. Ezup Column Bacteria Genomic DNA Purification Kit (B518255) was from Sangon Biotech (Shanghai) Co., Ltd. Premix Taq™ (R004A), DL2000 DNA Marker (3427 A) were purchased from TakaRa Bio Co., Ltd. High performance liquid chromatography (HPLC) grade methanol was purchased from Honeywell. Analytical pure organic reagents (methanol, ethyl acetate, dichloromethane, petroleum ether, anhydrous ethanol) were purchased from Guangdong Guanghua Technology Co., Ltd.

### Identification of the ***Achromobacter*** WA5-4-31

159 strains of intestinal bacteria were isolated from the *Periplaneta americana* and tested for their anti-*K. pneumoniae* activity by the Oxford Cup method. The secondary metabolites of WA5-4-31 showed strong activity against *K. pneumoniae*. The WA5-4-31 was inoculated on Gauze’s medium No.1 and incubate at 28 °C for 3 days. When the colony grew stably, the single colony was selected and cultivated in the ISP-1 seed medium at 160 rpm at 28 °C for 2 days. The morphology and surface characteristics of the strain WA5-4-31 were examined using a scanning electron microscope (FEI Phenom Desktop SEM, USA).

The DNA of WA5-4-31 was extracted by Ezup Column Bacteria Genomic DNA Purification Kit, and selected bacterial universal primers to amplify 16 S rDNA by PCR. The PCR was performed in a total volume of 20µL containing 10µL Premix Taq Mix, 2µL DNA template, 1µL primer 27f, 1µL primer 1492r, and 6µL ddH_2_O. Amplification conditions include pre-denaturation at 95 °C for 3 min, denaturation at 95 °C for 30s, annealing at 55 °C for 30s, and extension at 72 °C for 1 min, a total of 30 cycles, followed by extension at 72 °C for 10 min, and storage at 4 °C. The PCR products were verified by electrophoresis on a 1% agarose gel and sequenced in BGI. Finally the sequence were uploaded to National Center for Biotechnology Information (NCBI) and compared to all sequences available in GenBank. The phylogenetic tree was constructed using the neighbor joining method in the MEGA5.0 software [39].

### Fermentation, extraction and isolation

A 9 mL of seed culture was inoculated into a 500-mL Erlenmeyer flask containing 300 mL of the ISP 2 medium and incubated on rotary shakers (160 rpm) at 28℃. After 9 days, the fermentation broth was centrifuged at 5000rmp for 20 min. Then the supernatant of the fermentation broth was extracted with ethyl acetate(V:V = 1:1.5) for three times. After rotary evaporation and drying, 18 g crude ethyl acetate extract was obtained from 60 L of culture broth. Finally 9 fractions were obtained by thin layer chromatography and silica gel column gradient. Every fraction was tested for antimicrobial activity against *K. pneumoniae* [40] by Oxford Cup method. The active fractions were further purified by ODS column chromatography, Sephadex LH-20 column, and semi-prepared HPLC system(Waters e2535-2489, USA) with YMC-Pack ODS-AQ C18 column (250 × 10.0 mm, YMC AQ12S05-2546WT, Japan) using a UV-VIS detector and a 200µL injection loop at 25 ℃. The mobile phase was deionized water and methanol (H2O: methanol 100:0–0:100, V/V) at 1.0 mL/min over 65 min, and UV detection was recorded at λ = 280 nm.

### Spectroscopic analysis

The purity of compounds was analyzed using analytical HPLC with photodiode array detector (PAD) and YMC-Pack (ODS-AQ C18) column. Compounds were dissolved in DMSO. The dissolved Compounds were aspirated with a disposable syringe and filtered with 0.22 μm organic filter membrane for impurities. The proton and carbon nuclear magnetic resonance (NMR) spectra were recorded at 600 MHz using Brucker AVANCE III 600 M spectrometer (Brucker, Germany). The compounds were dissolved in methanol to prepare the concentration of 1 mg/L. Then the dissolved compounds were aspirated using a disposable syringe and filtered with 0.22 μm organic filter membrane for impurities. Mass spectra was obtained within the range of m/z 50-2000 by TSQ Endura™ Triple-quadrupole mass spectrometer (Thermo Fisher, USA). The molecular weight of the compounds was determined using mass spectrometry and their structure were characterized by 1 H NMR and 13 C NMR, combined with published literature.

### Determination of minimum inhibitory concentration (MIC)

The minimum inhibitory concentration (MIC) of purified compounds against the *K. pneumoniae* were determined by the tube-dilution method using individually pack-aged, flat bottomed, 96-well microtiter plates. The tested bacterial strain was cultivated in Mueller-Hinton Broth at 37 °C until the density reached approximately 1 ~ 5 × 10^5^ CFU/mL. Each of the tested compounds and drugs were dissolved in methanol and then diluted with sterile broth by the twofold dilution method. The final concentrations of each sample in the wells were 512, 256, 128, 64, 32, 16, 8, 4, 2, 1, 0.5 and 0.25 µg/mL. Ciprofloxacin and Ampicillin were used as positive control for *K. pneumoniae and S. aureus* respectively. A serial dilution of compounds were performed in the microplates and incubated at 37 °C for 24 h. All the experiment was performed in triplicate.

### Antibacterial activity assay

The activity of purified compounds against the *K. pneumoniae* were further determined by Oxford cup method. The tested bacterial strain was diluted to approximate 10^6^ CFU/mL, and mixed into LB medium (1% agar) to reach 0.5% (v/v) concentration. The mixture (15 mL) was poured into a LB plate with a sterilized Oxford cup placed up still in the plate. After cooling, the Oxford cups were taken out carefully to form round holes. Each tested compounds (50µL) were added to the small round holes. Ciprofloxacin was used as positive control. Methanol solvent was used as the control group. Plates were incubated at 37 °C for 24 h and photoed by Gel Imaging System (Gel DocTMXR+, Bio-Rad). The experiment was performed in triplicate, and the mean of the diameter of the inhibition zones was calculated.

### Scanning Electron Microscopy Analysis

The tested *K. pneumoniae* (1.5 × 10^8^ CFU/mL) was treated with 4 × MIC concentration of the compound at 37 °C for 24 h. The supernatant was removed by centrifuged and then the tested strain were placed on a cover glass and fixed overnight with 2.5% glutaraldehyde at 4 °C. Fixed samples were washed 3 times in 1x phosphate buffered saline (PBS) for 20 min respectively and dehydrated in gradient concentrations of ethanol (20%, 40%, 60%, 80%, and 100%). Finally the morphological changes of the tested *K. pneumoniae* was observed by Scanning Electron Microscopy (SEM) (Hitachi FlexSEM 1000, Japan) at an accelerating voltage of 2–19 kV.

## Electronic supplementary material

Below is the link to the electronic supplementary material.

**Supplementary Figures:** Mass spectrometry?Proton (1H) nuclear magnetic resonance and Carbon (13C) nuclear magnetic resonance spectrum of the compound 1-6

**Supplementary Tables:** NMR data of Compound 1-6

## Data Availability

All data generated or analyzed during this study are included in this published article and its supplementary information files.

## Abbreviations

HPLC: High Performance Liquid Chromatography
ODS: Octadecylsilyl
NMR: Nuclear Magnetic Resonance
HR-ESI-MS: High-resolution electrospray ionization mass spectroscopy
MEGA: Molecular Evolutionary Genetics Analysis
DMSO: Dimethyl Sulfoxide
CFU: Colony-forming Unit
CDCl3: Deuterated chloroform
m/z: Mass charge ratio.

## References

[1] Antimicrobial Resistance Collaborators (2022). Global burden of bacterial antimicrobial resistance in 2019: a systematic analysis. Lancet.

[2] Zhan Q, Xu Y, Wang B (2021). Distribution of fluoroquinolone resistance determinants in Carbapenem-resistant Klebsiella pneumoniae clinical isolates associated with bloodstream infections in China. BMC Microbiol.

[3] Šuto S, Bedenić B, Likić S (2022). Diffusion of OXA-48 carbapenemase among urinary isolates of Klebsiella pneumoniae in non-hospitalized elderly patients. BMC Microbiol.

[4] Lou T, Du X, Zhang P (2022). Risk factors for infection and mortality caused by carbapenem-resistant *Klebsiella pneumoniae*: a large multicentre case-control and cohort study. J Infect.

[5] Wang M, Earley M, Chen L (2022). Clinical outcomes and bacterial characteristics of carbapenem-resistant *Klebsiella pneumoniae* complex among patients from different global regions (CRACKLE-2): a prospective, multicentre, cohort study. LANCET INFECT DIS.

[6] Tarlachkov SV, Epiktetov DO, Sviridov AV et al. Draft genome sequence of glyphosate-degrading Achromobacter insolitus strain kg 19 (VKM B-3295), isolated from Agricultural Soil. Microbiol Resource Announcements. 2020, 9(17). 10.1128/MRA.00284-20 PMC7180283 32327510

[7] Sun L, Zhang X, Ouyang W (2022). Lowered cd toxicity, uptake and expression of metal transporter genes in maize plant by ACC deaminase-producing bacteria Achromobacter sp. J HAZARD MATER.

[8] Kuncharoen N, Muramatsu Y, Shibata C (2017). Achromobacter aloeverae sp. nov. isolated from root of Aloe vera (L.) Burm.f. INT J SYST EVOL MICR.

[9] Bing X, Winkler J, Gerlach J (2021). Identification of natural pathogens from wild Drosophila suzukii. Pest Manag Sci.

[10] Liang DH, Hu Y (2021). Application of a heavy metal-resistant Achromobacter sp. for the simultaneous immobilization of cadmium and degradation of sulfamethoxazole from wastewater. J HAZARD MATER.

[11] Subudhi S, Batta N, Pathak M (2014). Bioflocculant production and biosorption of zinc and lead by a novel bacterial species, Achromobacter sp. TERI-IASST N, isolated from oil refinery waste. Chemosphere.

[12] Deng Z, Jiang Y, Chen K (2020). One biosurfactant-producing Bacteria Achromobacter sp. A-8 and its potential use in Microbial enhanced oil recovery and bioremediation. Front Microbiol.

[13] Joy S, Rahman P, Khare SK (2019). Production and characterization of glycolipid biosurfactant from Achromobacter sp. (PS1) isolate using one-factor-at-a-time (OFAT) approach with feasible utilization of ammonia-soaked lignocellulosic pretreated residues. Bioprocess Biosyst Eng.

[14] Wang P, Gao J, Zhao Y (2021). Biodegradability of di-(2-ethylhexyl) phthalate by a newly isolated bacterium Achromobacter sp. RX SCI TOTAL ENVIRON.

[15] Chen ZY, Ou PY, Liu LY (2018). Isolation, identification and antibacterial activity of *Achromobacter sp* from *Periplaneta americana*. Chin J Zoonoses.

[16] Zeng C, Liao Q, Hu Y (2019). The role of Periplaneta americana (Blattodea: Blattidae) in modern Versus Traditional Chinese Medicine. J Med Entomol.

[17] Nguyen T, Chen X, Chai J (2020). Antipyretic, anti-inflammatory and analgesic activities of Periplaneta americana extract and underlying mechanisms. Biomed Pharmacother.

[18] Lin SS, Liu CX, Wang XL (2019). Intervention mechanisms of Xinmailong Injection, a Periplaneta Americana Extract, on Cardiovascular Disease: a systematic review of Basic Researches. Evidence-Based Complement Altern Med.

[19] Li LJ, Xu XH, Yuan TJ (2019). Periplaneta Americana L. as a novel therapeutics accelerates wound repair and regeneration. Biomed Pharmacother.

[20] Chen ZY, Ou PY, Liu LY, Jin XB (2020). Anti-MRSA activity of actinomycin X2 and collismycin a produced by Streptomyces globisporus WA5-2-37 from the intestinal tract of american Cockroach (Periplaneta americana). Front Microbiol.

[21] Xia F, Shen J, Jie W (2018). Antifungal activity of 3-acetylbenzamide produced by actinomycete WA23-4-4 from the intestinal tract of Periplaneta americana[J]. J Microbiol.

[22] Lin PB, Shen J, Ou PY (2019). Prodigiosin isolated from Serratia marcescens in the Periplaneta americana gut and its apoptosis- inducing activity in HeLa cells. Oncol Rep.

[23] Ma Y, Xu M, Liu H (2021). Antimicrobial compounds were isolated from the secondary metabolites of Gordonia, a resident of intestinal tract of Periplaneta americana. AMB Express.

[24] Challinor VL, BodeH B (2015). Bioactive natural products from novel microbial sources. Ann N Y Acad Sci.

[25] Zothanpuia, Passari AK, Gupta VK (2016). Detection of antibiotic-resistant bacteria endowed with antimicrobial activity from a freshwater lake and their phylogenetic affiliation. PeerJ.

[26] Vijay K, Devi TS, Sree KK (2020). In vitro screening and in silico prediction of antifungal metabolites from rhizobacterium Achromobacter kerstersii JKP9. ARCH MICROBIOL.

[27] Lim HJ, An JS, Bae ES, et al. Ligiamycins a and B, decalin-amino-maleimides from the co-culture of Streptomyces sp. and Achromobacter sp. Isolated from the Marine Wharf Roach, Ligia exotica. Mar Drugs. 2022;20(2). 10.3390/md20020083 10.3390/md20020083 PMC8878407 35200613

[28] Deepa I, Kumar SN, Sreerag RS (2015). Purification and synergistic antibacterial activity of arginine derived cyclic dipeptides, from Achromobacter sp. associated with a rhabditid entomopathogenic nematode against major clinically relevant biofilm forming wound bacteria. Front Microbiol.

[29] Shahsavari N, Wang B, Imai Y (2022). A Silent Operon of Photorhabdus luminescens encodes a Prodrug Mimic of GTP. mBio.

[30] Dahal RH, Nguyen TM, Pandey RP (2020). The genome insights of Streptomyces lannensis T1317-0309 reveals actinomycin D production. J ntibiotics.

[31] dagawa T, Yuan J, Panigrahy D (2000). Cytochalasin E, an epoxide containing aspergillus-derived fungal metabolite, inhibits angiogenesis and tumor growth. J Pharmacol Experimental Ther.

[32] Sharma M, Manhas RK (2019). Purification and characterization of actinomycins from Streptomyces strain M7 active against methicillin resistant Staphylococcus aureus and vancomycin resistant Enterococcus. BMC Microbiol.

[33] Koba M, Konopa J (2005). Actinomycin D and its mechanisms of action. Postepy Hig Med Dosw.

[34] Zeng H, Feng PX, Wan CX (2018). Antifungal effects of actinomycin D on Verticillium dahliae via a membrane-splitting mechanism[J]. Nat Prod Res.

[35] Wu HC, Rérolle D, Berthier C (2021). Actinomycin D targets NPM1c-primed mitochondria to restore PML-driven senescence in AML therapy. CANCER DISCOV.

[36] Zeng YX, Liu JS, Wang YJ (2022). Actinomycin D: a novel Pseudomonas aeruginosa quorum sensing inhibitor from the endophyte Streptomyces cyaneochromogenes RC1. WORLD J MICROB BIOT.

[37] Yao G, Chen X, Zheng H, et al. Genomic and Chemical Investigation of Bioactive secondary metabolites from a Marine-Derived Fungus Penicillium steckii P2648. Front Microbiol. 2021-01-01;12:600991. 10.3389/fmicb.2021.600991 PMC8211754 34149630

[38] Delebassée S, Mambu L, Pinault E (2017). Cytochalasin E in the lichen Pleurosticta acetabulum. Anti-proliferative activity against human HT-29 colorectal cancer cells and quantitative variability. Fitoterapia.

[39] Tamura K, PetersonD, Peterson N (2011). MEGA5: Molecular Evolutionary Genetics Analysis using Maximum Likelihood, Evolutionary Distance, and Maximum Parsimony Methods. Mol Biology Evol.

[40] Esposito M, Nothias LF, Nedev H (2016). Euphorbia dendroides latex as a source of Jatrophane Esters: isolation, structural analysis, conformational study, and Anti-CHIKV activity. Nat Prod.

