# Supplementary material for: Anti-Klebsiella pneumoniae activity of secondary metabolism of Achromobacter from the intestine of Periplaneta americana
Source: BMC Microbiol. 2023 Jun 5;23:162. doi: 10.1186/s12866-023-02909-7 (PMC10240788; doi:10.1186/s12866-023-02909-7)
Supplement: Supplementary file 1 — Supplementary Figures: Mass spectrometry?Proton (1H) nuclear magnetic resonance and Carbon (13C) nuclear magnetic resonance spectrum of the compound 1-6 [file 12866_2023_2909_MOESM1_ESM.docx]

1. ESI-MS:

1. ^1^H NMR:


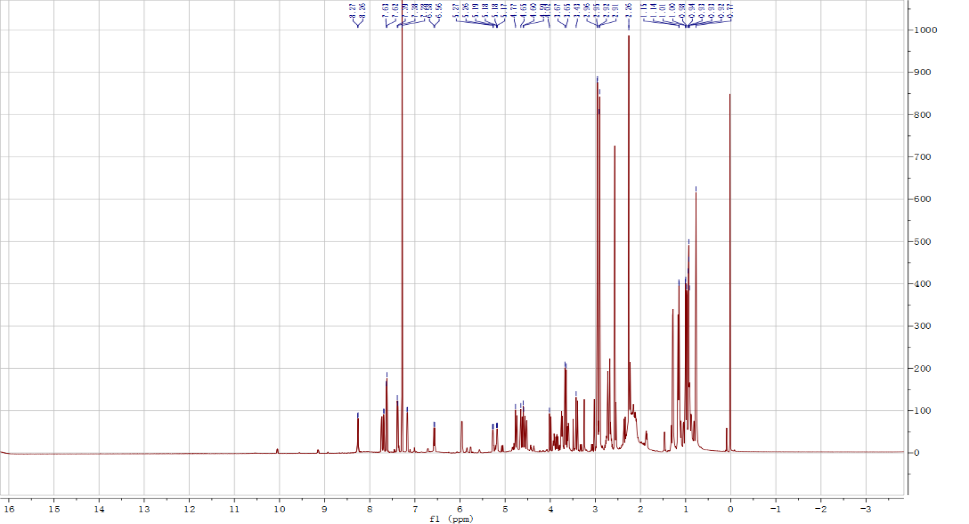


1. ^13^C NMR:


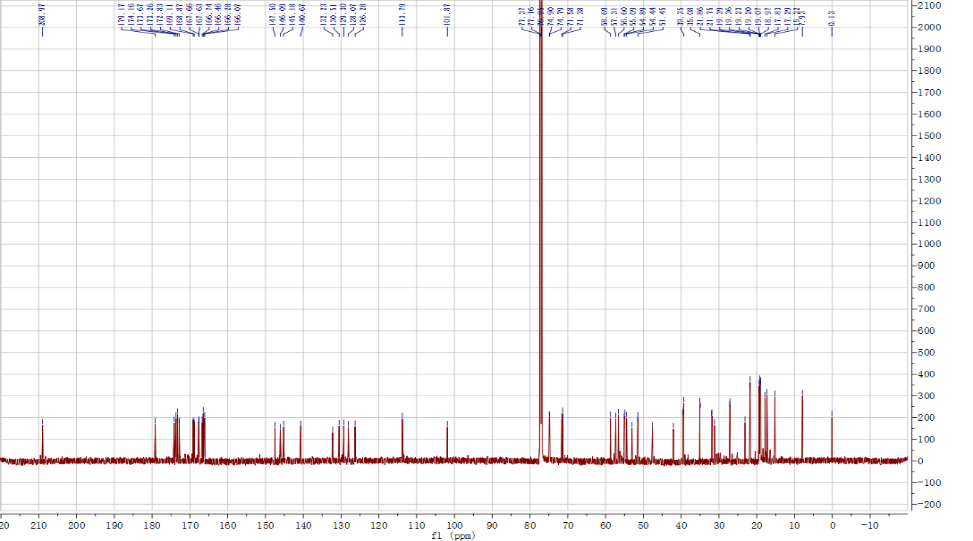


**Figure S1 Identification of compound 1: Actinomycin D**

(a) Mass spectrometry. (b) Proton (1H) nuclear magnetic resonance. (c) Carbon (13C) nuclear magnetic resonance

(a) ESI-MS:

(b) ^1^H NMR:


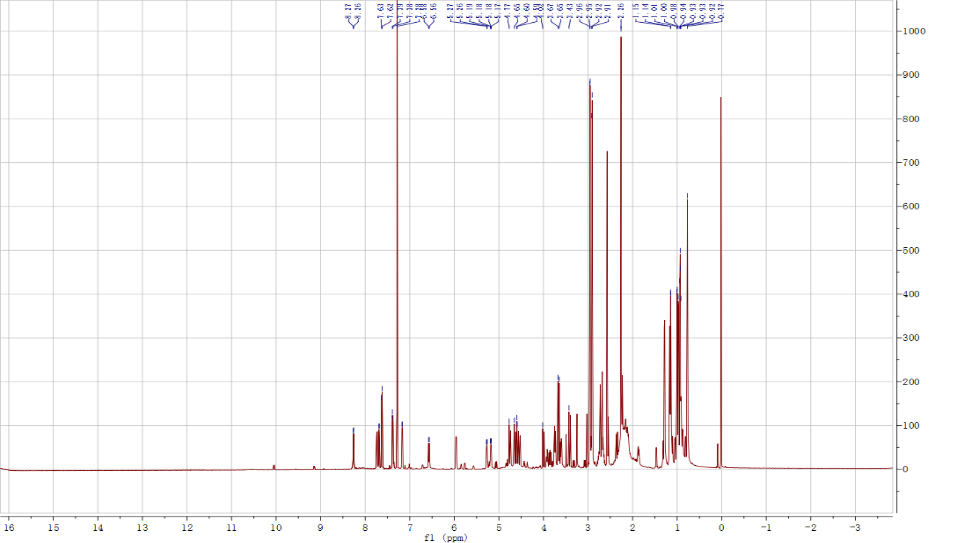


(c)^13^C NMR:


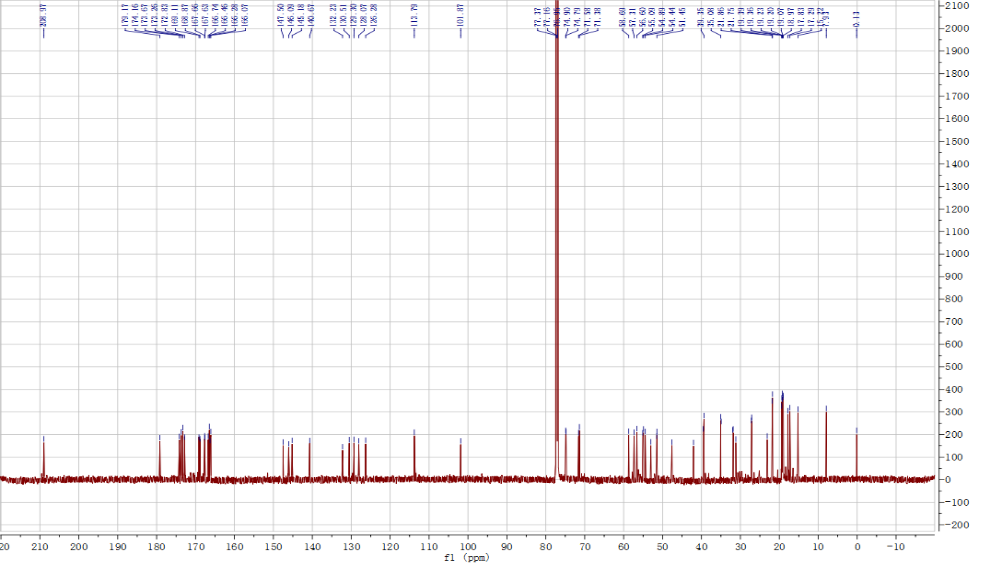


**Figure S2 Identification of compound 2: Actinomycin X_2_**

1. Mass spectrometry. (b) Proton (1H) nuclear magnetic resonance. (c) Carbon (13C) nuclear magnetic resonance

(a) ESI-MS:

(b) ^1^H NMR:


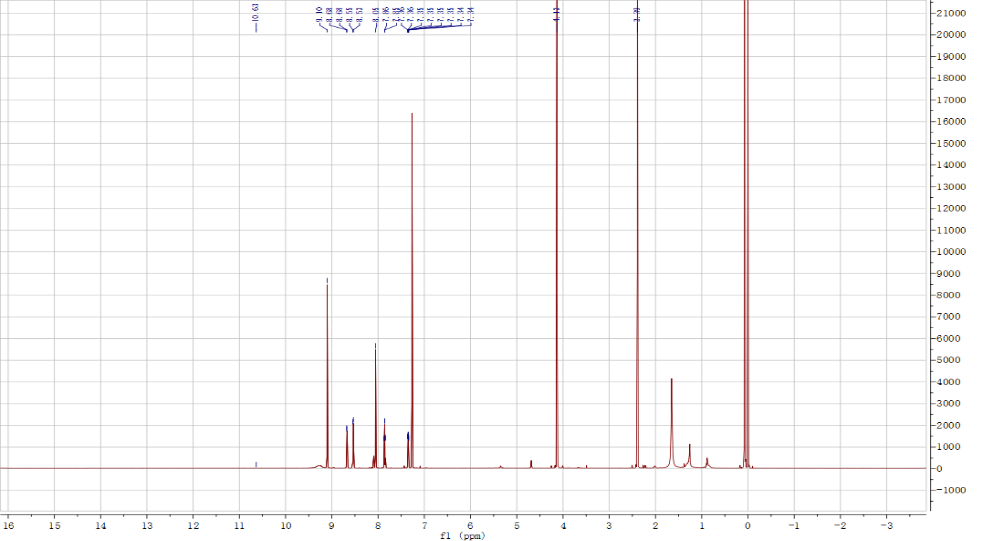


(c) ^13^C NMR:


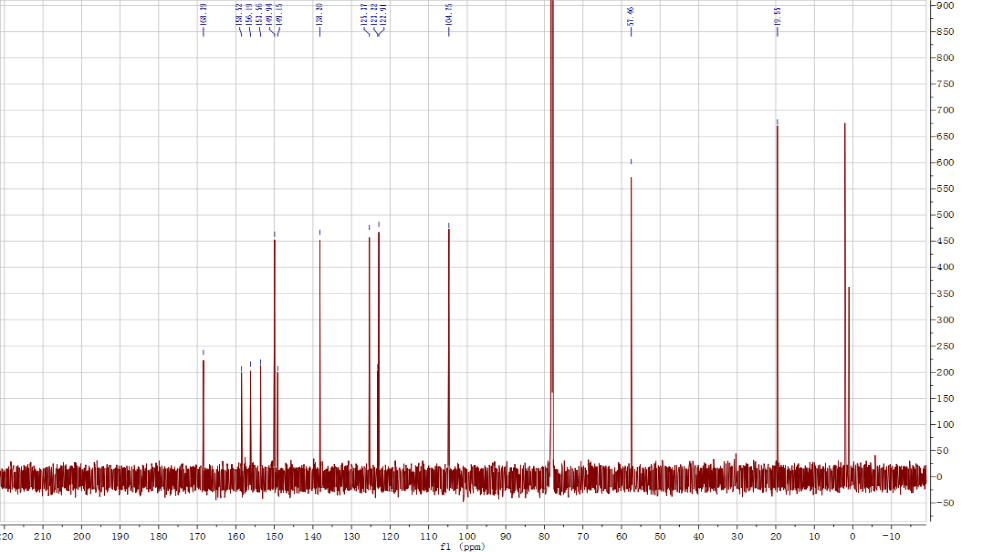


**Figure S3 Identification of compound 3: CollismycinA**

1. Mass spectrometry. (b) Proton (1H) nuclear magnetic resonance. (c) Carbon (13C) nuclear magnetic resonance
2. ESI-MS:

1. ^1^H NMR:


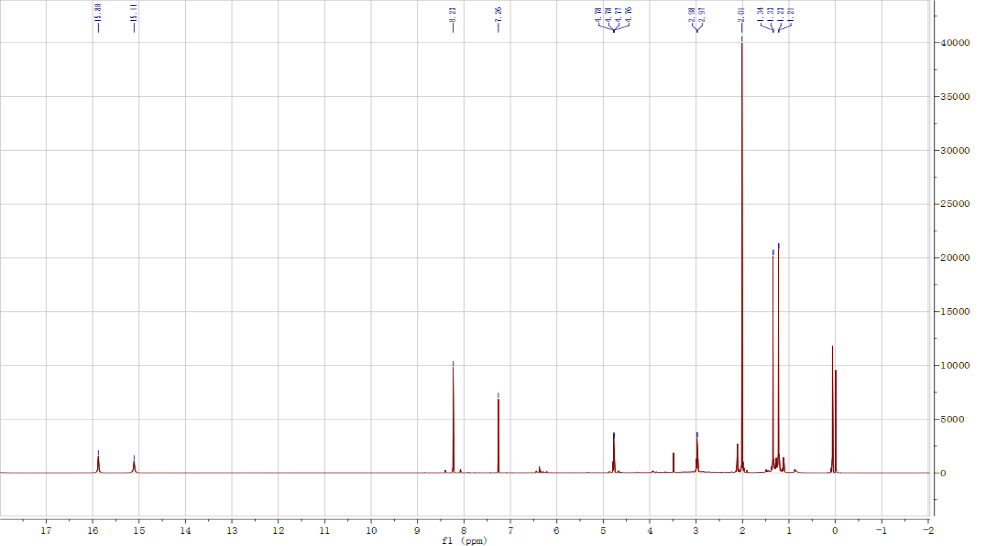


(c)^13^C NMR:


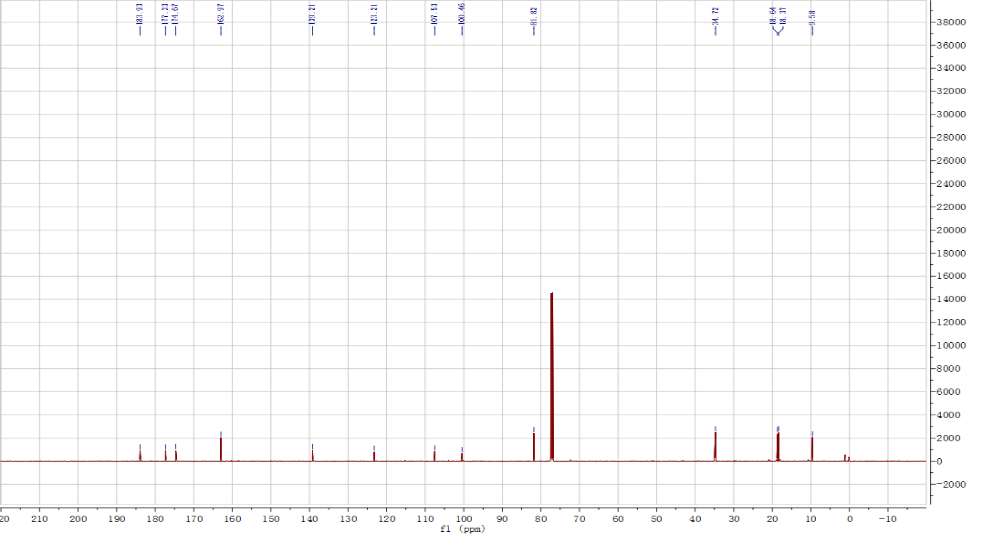


**Figure S4 Identification of compound 4: Citrinin**

(a) Mass spectrometry. (b) Proton (1H) nuclear magnetic resonance. (c) Carbon (13C) nuclear magnetic resonance

1. ESI-MS:

1. ^1^H NMR:


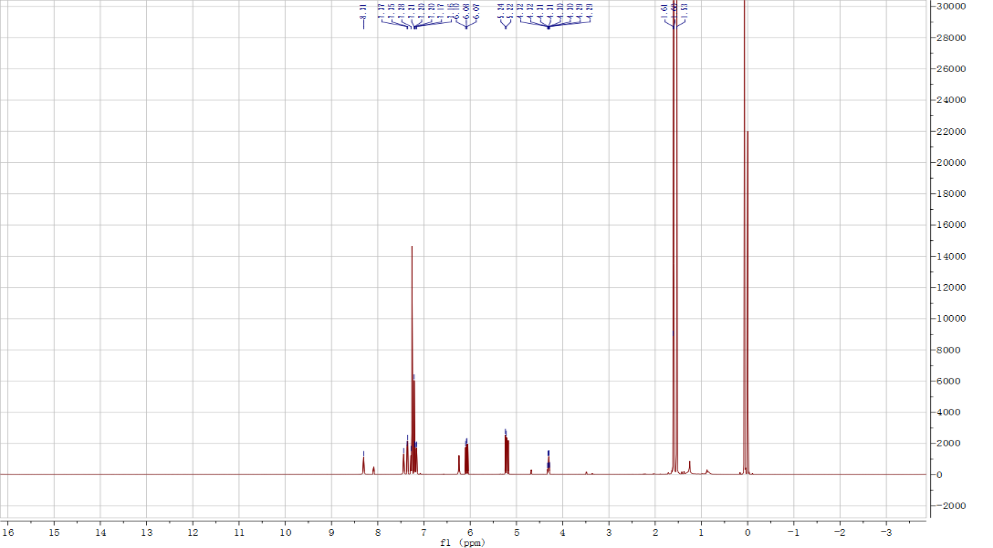


(c)^13^C NMR:


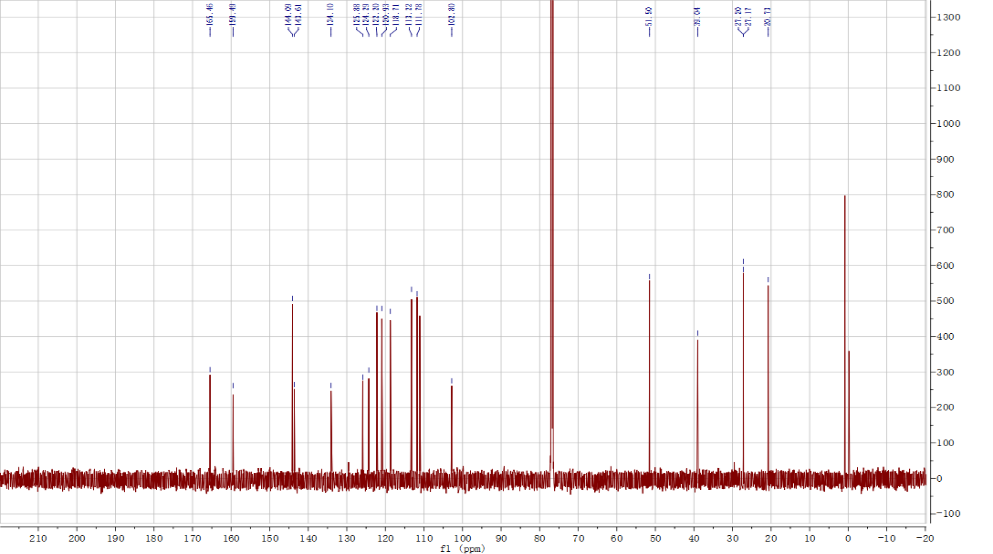


**Figure S5 Identification of compound 5: Neoechinulin A**

1. Mass spectrometry. (b) Proton (1H) nuclear magnetic resonance. (c) Carbon (13C) nuclear magnetic resonance
2. ESI-MS:

1. ^1^H NMR:


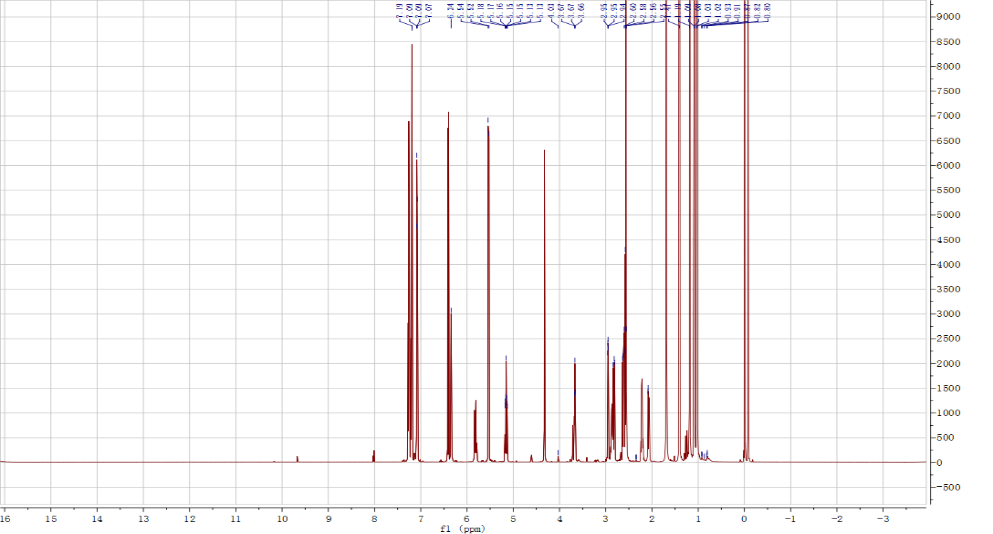


(c)^13^C NMR:


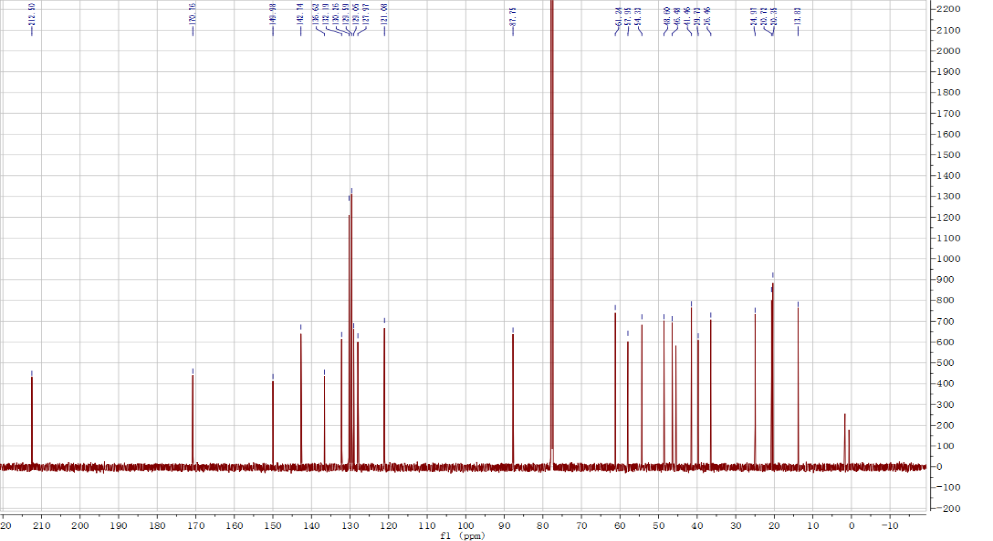


**Figure S6 Identification of compound 6: Cytochalasin E**

(a) Mass spectrometry. (b) Proton (1H) nuclear magnetic resonance. (c) Carbon (13C) nuclear magnetic resonance
