# Supplementary material for: Anti-Klebsiella pneumoniae activity of secondary metabolism of Achromobacter from the intestine of Periplaneta americana
Source: BMC Microbiol. 2023 Jun 5;23:162. doi: 10.1186/s12866-023-02909-7 (PMC10240788; doi:10.1186/s12866-023-02909-7)
Supplement: Supplementary file 2 — Supplementary Tables: NMR data of Compound 1-6 [file 12866_2023_2909_MOESM2_ESM.docx]

**Table S1 NMR data correlations of Compound 1 wih Actinomycin D (CDCl_3_)**

| C^#^ | Compound 1 | | Actinomycin D | |
| --- | --- | --- | --- | --- |
|  | ^1^H NMR | ^13^C NMR | ^1^H NMR | ^13^C NMR |
| 1 |  | 101.81 |  | 101.70 |
| 2 |  | 147.71 |  | 147.70 |
| 3 |  | 179.17 |  | 179.10 |
| 4 |  | 113.69 |  | 113.50 |
| 6 |  | 127.86 |  | 127.80 |
| 7 | 7.32(1H,d) | 130.44 | 7.35(1H,d) | 130.40 |
| 8 | 7.58(1H,d) | 125.79 | 7.60(1H,d) | 125.80 |
| 9 |  | 132.62 |  | 132.60 |
| 11 | 2.64(3H,s) | 15.17 | 2.69(3H,s) | 15.10 |
| 12 | 2.51(3H,s) | 7.88 | 2.54(3H,s) | 7.80 |
| 13 |  | 166.71 |  | 166.70 |
| 14 |  | 169.14 |  | 169.10 |
| 4a |  | 145.19 |  | 145.10 |
| 5a |  | 140.62 |  | 140.50 |
| 9a |  | 129.19 |  | 129.20 |
| 10a |  | 145.96 |  | 145.90 |
| L-Thr1 |  | 168.65 |  | 168.70 |
| 2 | 4.61(1H,d) | 55.27 | 4.63(1H,d) | 55.20 |
| 3 | 5.19(1H,qd) | 75.16 | 5.21(1H,qd) | 75.10 |
| 4 | 1.24(3H,d) | 17.83 | 1.25(3H,d) | 17.80 |
| D-Val1 |  | 173.73 |  | 173.70 |
| 2 |  | 58.98 |  | 58.90 |
| 3 |  | 31.89 |  | 31.80 |
| 4 | 1.11(3H,d) | 19.39 | 1.11(3H,d) | 19.40 |
| 5 | 0.86(6H,d) | 19.14 | 0.89(6H,d) | 19.10 |
| L-Pto1 |  | 173.37 |  | 173.40 |
| 2 | 2.91(3H,s) | 56.64 | 2.89(3H,s) | 56.60 |
| 3 |  | 31.36 |  | 31.30 |
| 4 |  | 23.11 |  | 23.10 |
| 5 |  | 47.70 |  | 47.70 |
| Sar1 |  | 166.58 |  | 166.50 |
| 2 | 3.64(2H,d) | 51.48 | 3.66(2H,d) | 51.50 |
| N-CH3 | 2.90(3H,s) | 35.06 | 2.89(3H,s) | 35.00 |
| L-Mevl |  | 167.80 |  | 167.80 |
| 2 |  | 71.46 |  | 71.40 |
| 3 |  | 27.09 |  | 27.00 |
| 4 | 0.94(3H,d) | 21.77 | 0.95(3H,d) | 21.70 |
| 5 | 0.73(6H,d) | 19.35 | 0.74(6H,d) | 19.30 |
| N-CH3 | 2.92(3H,s) | 39.39 | 2.92(3H,s) | 39.30 |
| L-Thr1 |  | 167.71 |  | 167.70 |
| 2 | 4.51(1H,dd) | 54.96 | 4.52(1H,dd) | 54.90 |
| 3 | 5.20(1H,qd) | 75.01 | 5.21(1H,qd) | 75.00 |
| 4 | 1.23(3H,d) | 17.42 | 1.23(3H,d) | 17.40 |
| D-Val1 |  | 173.41 |  | 173.40 |
| 2 |  | 58.81 |  | 58.80 |
| 3 |  | 31.65 |  | 31.60 |
| 4 | 1.11(3H,d) | 19.20 | 1.10(3H,d) | 19.20 |
| 5 | 0.87(6H,d) | 19.09 | 0.89(6H,d) | 19.10 |
| Pto1 |  | 173.43 |  | 173.30 |
| 2 | 2.86(3H,s) | 56.42 | 2.87(3H,s) | 56.40 |
| 3 |  | 31.05 |  | 31.00 |
| 4 |  | 22.94 |  | 22.90 |
| 5 |  | 47.47 |  | 47.40 |
| Sar1 |  | 166.44 |  | 166.40 |
| 2 | 3.62(2H,d) | 51.41 | 3.66(2H,d) | 51.40 |
| N-CH3 | 2.90(3H,s) | 35.00 | 2.89(3H,s) | 35.00 |
| L-Mev1 |  | 166.64 |  | 166.60 |
| 2 |  | 71.31 |  | 71.20 |
| 3 |  | 27.08 |  | 27.00 |
| 4 | 0.93(3H,d) | 21.61 | 0.91(3H,d) | 21.60 |
| 5 | 0.75(6H,d) | 19.20 | 0.74(6H,d) | 19.10 |
| N-CH3 | 2.82(3H,s) | 39.28 | 2.87(3H,s) | 39.20 |

**Table S2 NMR data correlations of Compound 2 with Actinomycin X_2_(CDCl_3_)**

| C^#^ | **Compound 2** | | **Actinomycin X_2_** | |
| --- | --- | --- | --- | --- |
|  | ^1^H NMR | ^13^C NMR | ^1^H NMR | ^13^C NMR |
| 1 |  | 101.82 |  | 101.66 |
| 2 |  | 147.55 |  | 147.34 |
| 3 |  | 179.12 |  | 178.98 |
| 4 |  | 113.70 |  | 113.61 |
| 5 |  | 146.03 |  | 145.90 |
| 6 |  | 140.61 |  | 140.48 |
| 7 |  | 128.10 |  | 127.91 |
| 8 | 7.32(1H,d) | 130.55 | 7.35(1H,d) | 130.34 |
| 9 | 7.65(1H,d) | 126.24 | 7.59(1H,d) | 126.10 |
| 10 |  | 132.21 |  | 132.02 |
| 11 |  | 129.32 |  | 129.12 |
| 12 |  | 145.15 |  | 125.00 |
| 4-CH_3_ | 2.20(3H,s) | 7.95 | 2.21(3H,s) | 7.75 |
| 7-CH_3_ | 2.52(3H,s) | 15.29 | 2.53(3H,s) | 15.06 |
| 1-CO |  | 166.71 |  | 166.75 |
| 10-CO |  | 166.28 |  | 165.90 |
| α-ring |  | 168.87 |  | 168.69 |
| L-Thr1 |  |  |  |  |
| -NH | 7.14(1H,d) |  | 7.15(1H,d) |  |
| 2 | 4.57(1H,dd) | 55.09 | 4.55(1H,dd) | 54.92 |
| 3 | 5.16(1H,qd) | 74.79 | 5.14(1H,qd) | 74.73 |
| 4 | 1.12(3H,d) | 17.29 | 1.11(3H,d) | 17.12 |
| D-Val1 |  | 173.67 |  | 173.48 |
| -NH | 7.71(1H,d) |  | 7.69(1H,d) |  |
| 2 | 3.58(1H,dd) | 58.69 | 3.56(1H,dd) | 58.51 |
| 3 | 2.10(1H,m) | 31.85 | 2.07(1H,m) | 31.83 |
| 4 | 0.90(3H,d) | 18.97 | 0.89(3H,d) | 18.90 |
| 5 | 1.12(3H,d) | 19.20 | 1.09(3H,d) | 19.36 |
| L-Pro1 |  | 173.26 |  | 173.08 |
| 2 | 5.93(1H,d) | 56.60 | 5.92(1H,d) | 56.43 |
| 3 | 1.85(1H,dd)2.65  (1H,m) | 31.17 | 1.85(1H,dd)  2.67(1H,m) | 31.00 |
| 4 | 2.08(1H,m)2.22  (1H,m) | 23.12 | 2.08(1H,m)  2.22(1H,m) | 22.95 |
| 5 | 3.72(1H,m)3.87  (1H,m) | 47.59 | 3.72(1H,m)  3.87(1H,m) | 47.72 |
| Sar1 |  | 166.46 |  | 166.28 |
| N-CH_3_ | 2.88(3H,s) | 34.93 | 2.86(3H,s) | 34.76 |
| 2 | 3.62(1H,d),4.73  (1H,d) | 51.45 | 3.62(1H,d)  4.71(1H,d) | 51.31 |
| L-Mev1 |  | 167.63 |  | 167.48 |
| N-CH_3_ | 2.94(3H,s) | 39.53 | 2.91(3H,s) | 39.68 |
| 2 | 2.67(1H,m) | 71.38 | 2.66(1H,m) | 71.19 |
| 3 | 2.67(1H,m) | 27.10 | 2.66(1H,m) | 26.93 |
| 4 | 0.72(3H,d) | 19.26 | 0.72(3H,d) | 19.19 |
| 5 | 0.96(3H,d) | 21.65 | 0.93(3H,d) | 21.58 |
| β-ring |  | 169.11 |  | 168.93 |
| L-Thr1 |  |  |  |  |
| -NH | 7.68(1H,d) |  | 7.65(1H,d) |  |
| 2 | 4.51(1H,dd) | 54.82 | 4.47(1H,dd) | 54.71 |
| 3 | 5.24(1H,qb) | 74.70 | 5.23(1H,qb) | 74.62 |
| 4 | 1.25(3H,d) | 17.73 | 1.24(3H,d) | 17.62 |
| D-Val1 |  | 174.16 |  | 173.98 |
| -NH | 8.24(1H,d) |  | 8.22(1H,d) |  |
| 2 | 3.72(1H,dd) | 57.17 | 3.69(1H,dd) | 57.17 |
| 3 | 2.03(1H,m) | 32.80 | 2.07(1H,m) | 31.84 |
| 4 | 0.84(3H,d) | 19.07 | 0.87(3H,d) | 19.03 |
| 5 | 1.15(3H,d) | 19.32 | 1.13(3H,d) | 19.40 |
| L-4-Opr1 |  | 172.70 |  | 172.65 |
| 2 | 6.54(1H,d) | 54.34 | 6.53(1H,d) | 54.27 |
| 3 | 2.32(1H,d)  3.83(1H,dd) | 42.01 | 2.31(1H,d)  3.81(1H,dd) | 41.88 |
| 4 |  | 208.97 |  | 208.81 |
| 5 | 3.89(1H,d)4.57  (1H,d) | 52.92 | 3.96(1H,d)  4.54(1H,d) | 52.85 |
| Sar1 |  | 166.07 |  | 166.11 |
| N-CH_3_ | 2.84(3H,s) | 35.04 | 2.87(3H,s) | 34.91 |
| 2 | 3.66(1H,d)  4.55(1H,d) | 51.43 | 3.62(1H,d)  4.59(1H,d) | 51.29 |
| L-Mev1 |  | 167.65 |  | 167.50 |
| N-Me | 2.90(3H,s) | 31.84 | 2.92(3H,s) | 31.84 |
| 2 | 2.64(1H,m) | 71.40 | 2.66(1H,m) | 71.40 |
| 3 | 2.63(1H,m) | 27.15 | 2.66(1H,m) | 26.99 |
| 4 | 0.72(3H,d) | 19.31 | 0.73(3H,d) | 19.23 |
| 5 | 0.97(3H,d) | 21.80 | 0.95(3H,d) | 21.70 |

**Table S3 NMR data correlations of Compound 3 with CollismycinA(CDCl_3_)**

| C^#^ | Compound 3 | | CollismycinA | |
| --- | --- | --- | --- | --- |
|  | 1H NMR | 13C NMR | 1H NMR | 13C NMR |
| 2 |  | 158.52 |  | 157.40 |
| 3 | 8.03(1H,s) | 104.75 | 8.06(1H,s) | 103.70 |
| 4 |  | 168.39 |  | 167.40 |
| 5 |  | 123.22 |  | 122.10 |
| 6 |  | 153.56 |  | 152.60 |
| 7 | 9.09(1H,s) | 149.15 | 9.11(1H,s) | 147.60 |
| 2' |  | 156.19 |  | 155.20 |
| 3' | 8.52(1H,d) | 122.91 | 8.56(1H,d) | 121.90 |
| 4' | 7.85(1H,td) | 138.20 | 7.87(1H,td) | 137.20 |
| 5' | 7.35(1H,ddd) | 125.37 | 7.35(1H,ddd) | 124.30 |
| 6' | 8.68(1H,m) | 149.94 | 8.67(1H,m) | 148.90 |
| -OH | 10.63(1H,s) |  | 10.79(1H,s) |  |
| O-CH_3_ | 4.13(3H,s) | 57.46 | 4.12(3H,s) | 56.40 |
| S-CH_3_ | 2.39(3H,s) | 19.55 | 2.38(3H,s) | 18.50 |

**Table S4 NMR data correlations of Compound 4 with citrinin（CDCl_3_）**

| C^#^ | Compound 4 | | citrinin | |
| --- | --- | --- | --- | --- |
|  | ^1^H NMR | ^13^C NMR | ^1^H NMR | ^13^C NMR |
| 1 | 8.25(1H,s) | 162.97 | 8.23(1H,s) | 163.51 |
| 3 | 4.78(1H,q) | 81.82 | 4.77(1H,q) | 81.82 |
| 4 | 3.00(1H,q) | 34.72 | 2.98(1H,q) | 34.61 |
| 5 |  | 123.21 |  | 123.18 |
| 6 |  | 177.33 |  | 177.31 |
| 7 |  | 100.46 |  | 100.44 |
| 8 |  | 183.93 |  | 183.82 |
| 9 |  | 107.53 |  | 107.41 |
| 10 |  | 139.21 |  | 139.30 |
| 11 | 1.35(3H,d) | 18.64 | 1.33(3H,d) | 18.61 |
| 12 | 1.23(3H,d) | 18.37 | 1.21(3H,d) | 18.37 |
| 13 | 2.03(3H,s) | 9.58 | 2.00(3H,s) | 9.51 |
| 14 |  | 174.67 |  | 174.68 |
| -OH | 15.90(1H,s) |  | 15.90(1H,s) |  |
| -OH | 15.13(1H,s) |  | 15.11(1H,s) |  |

**Table S5 NMR data correlations of Compound 5 with** Neoechinulin A**(CDCl_3_)**

| C^#^ | Compound 5 | | Neoechinulin A | |
| --- | --- | --- | --- | --- |
|  | ^1^H NMR | ^13^C NMR | ^1^H NMR | ^13^C NMR |
| 1 | 8.30(1H,s) |  | 8.33(1H,s) |  |
| 2 |  | 143.61 |  | 143.90 |
| 3 |  | 102.80 |  | 103.14 |
| 3a |  | 125.88 |  | 126.21 |
| 4 | 7.27(1H,d) | 118.71 | 7.28(1H,d) | 119.03 |
| 5 | 7.19(1H,dd) | 120.93 | 7.20(1H,dd) | 121.21 |
| 6 | 7.17(1H,dd) | 122.20 | 7.16(1H,dd) | 122.52 |
| 7 | 7.36(1H,d) | 111.78 | 7.36(1H,d) | 111.49 |
| 7a |  | 134.10 |  | 134.54 |
| 8 | 7.20(1H,s) | 111.78 | 7.21(1H,s) | 112.11 |
| 9 |  | 124.29 |  | 124.70 |
| 10 |  | 159.49 |  | 160.01 |
| 11 | 7.43(1H,s) |  | 7.46(1H,s) |  |
| 12 | 4.30(1H,qd) | 51.50 | 4.31(1H,qd) | 51.82 |
| 13 |  | 165.46 |  | 165.90 |
| 14 | 6.57(1H,s) |  | 6.61(1H,s) |  |
| 15 |  | 39.04 |  | 39.45 |
| 16 | 6.07(1H,dd) | 144.09 | 6.08(1H,dd) | 144.52 |
| 17 | 5.23(1H,d)  5.17(1H,d) | 113.22 | 5.23(1H,d)  5.19(1H,d) | 113.55 |
| 18 | 1.52(6H,s) | 27.17 | 1.53(6H,s) | 27.51 |
| 19 | 1.52(6H,s) | 27.20 | 1.53(6H,s) | 27.52 |
| 20 | 1.59(3H,d) | 20.73 | 1.61(3H,d) | 21.00 |

**Table S6 NMR data correlations of Compound 6 with** Cytochalasin E(**CDCl_3_)**

| C# | **Compound 6** | | Cytochalasin E | |
| --- | --- | --- | --- | --- |
|  | ^1^H NMR | ^13^C NMR | ^1^H NMR | ^13^C NMR |
| 1 |  | 170.76 |  | 170.22 |
| 3 | 3.67(1H,br.s) | 54.33 | 3.66(1H,br.s) | 53.73 |
| 4 | 2.96(1H,br.s) | 48.60 | 3.01(1H,br.s) | 47.89 |
| 5 | 2.08(1H,m) | 36.46 | 1.98(1H,m) | 35.82 |
| 6 |  | 57.95 |  | 57.34 |
| 7 | 2.55(1H,m) | 61.24 | 2.48(1H,m) | 60.68 |
| 8 | 2.60(1H,d) | 46.48 | 2.59(1H,d) | 45.87 |
| 9 |  | 87.75 |  | 87.20 |
| 10 | 2.81(2H,m) | 45.50 | 2.76(2H,m) | 44.75 |
| 11 | 0.87(3H,d) | 13.83 | 0.88(3H,d) | 13.29 |
| 12 | 1.09(3H,s) | 20.35 | 1.11(3H,s) | 19.17 |
| 13 | 5.54(1H,m) | 129.05 | 5.74(1H,m) | 128.49 |
| 14 | 5.13(1H,m) | 130.26 | 5.04(1H,m) | 131.52 |
| 15 | 2.55(1H,m)  2.09(1H,m) | 39.73 | 2.48(1H,m)  2.09(1H,m) | 39.13 |
| 16 | 2.84(1H,m) | 41.46 | 2.88(1H,m) | 40.82 |
| 17 |  | 212.50 |  | 211.91 |
| 18 |  | 77.45 |  | 76.70 |
| 19 | 5.54(1H,d) | 121.08 | 5.43(1H,d) | 120.42 |
| 20 | 6.34(1H,d) | 142.74 | 6.21(1H,d) | 142.15 |
| 22 |  | 149.98 |  | 149.33 |
| 24 | 0.93(3H,m) | 20.72 | 0.99(3H,m) | 20.16 |
| 25 | 1.19(3H,s) | 24.97 | 1.29(3H,s) | 24.37 |
| 1’ |  | 136.62 |  | 135.92 |
| 2’  6’ | 7.01(2H,m) | 129.59 | 7.01(2H,m) | 129.72 |
| 3’  5’ | 7.19-7.07(1H,m) | 129.05 | 7.20-7.07(2H,m) | 128.91 |
| 4’ | 7.19-7.07(1H,m) | 127.97 | 7.20-7.07(1H,m) | 127.35 |
| NH | 5.15(1H,s) |  | 5.14(1H,s) |  |
| 18-OH | 4.03(1H,s) |  | 4.09(1H,s) |  |
